# Supplementary material for: Single-shot on-chip spectral sensors based on photonic crystal slabs
Source: Nat Commun. 2019 Mar 4;10:1020. doi: 10.1038/s41467-019-08994-5 (PMC6399238; doi:10.1038/s41467-019-08994-5)
Supplement: Supplementary file 1 — Supplementary Information [file 41467_2019_8994_MOESM1_ESM.pdf]

Supplementary Information for

"Single-shot on-chip spectral sensors based on photonic crystal slabs"

Wang et al.

Supplementary Note 1: Spectrum Reconstruction

The unknown spectrum $I(\lambda)$ is measured by recording its transmitted intensity through each PC structure by a CMOS sensor, with λ denoting the wavelength. Mathematically, for the ith PC, the measured signal S_i can be written as

$$S_i = \int I(\lambda)T_i(\lambda)Q(\lambda)\frac{\lambda}{\hbar c}\, d\lambda, \quad i = 1, 2, \cdots, M. \tag{1}$$

Here, $\hbar$ is reduced Planck constant, c is the speed of light in vacuum, and $T_i(\lambda)$ the transmission spectrum of the ith structure, which could be predetermined in the calibration process. The quantum efficiency of the sensor $Q(\lambda)$ is calculated as

$$Q(\lambda) = \frac{S_{\text{CMOS}}(\lambda)}{\frac{P_{\text{in}}(\lambda)\lambda}{\hbar c}}, \tag{2}$$

where $P_{\text{in}}(\lambda)$ is the power of the incident light at wavelength λ, $S_{\text{CMOS}}(\lambda)$ is the signal recorded by the CMOS sensor without PC slabs. Combining Eqs. (1) and (2), we get

$$S_i = \int I(\lambda)T_m(\lambda)\frac{S_{\text{CMOS}}(\lambda)}{P_{\text{in}}(\lambda)}\, d\lambda. \tag{3}$$

We then name $\eta(\lambda) = S_{\text{CMOS}}(\lambda)/P_{\text{in}}(\lambda)$, which is measured by taking the ratio of signal received by the CMOS sensor and a power meter illuminated by the same monochromatic light for each wavelength.

For recovery purposes, the unknown spectrum $I(\lambda)$ is discretized into a N-dimension vector $I_n = I(\lambda_n), n = 1, 2, \cdots, N$. Then Eq. (1) is turned into M linear equations with N unknowns

$$S = T\eta I. \tag{4}$$

We can find the input spectra I that minimizes the l_2 norm

$$||S - T\eta I||_2^2 \quad \text{subject to} \quad I_n > 0.$$

In practice, S is subject to experimental noise, then a smooth signal I that approximates S can be reconstructed as a solution to a regularized objective function

$$\min_I ||S - T\eta I||_2^2 + k||DI||_2^2 \quad \text{subject to} \quad I_n > 0, \tag{5}$$

where $k > 0$ is the weight. DI is the second-order derivative of the signal I [1, 2]. Minimizing $||DI||_2^2$ forces I to be smooth.

Supplementary Note 2: Structure Design

The proposed PC-spectrometer integrates an array of PC structures with a variety of periodicities and shapes. The parameters for each PC structure are chosen so that the spectral responsivity

is substantially different between the various PC slabs. In order to achieve this goal, we simulated the spectral responsivities for hundreds of PC structures, and selected ones that less correlated. The simulation was performed in S_4 [3] by solving Maxwells equations using rigorous coupled-wave analysis (RCWA).

To determine the number of structures needed for the recovery of an incident spectrum, we generated several spectra and simulated the measurement process with different numbers of independent PC slabs. In each test, the spectrum is digitalized with 1-nm resolution in a 200-nm spectral range, represented by 201 unknowns. In Supplementary Figure 1, we plot the reconstruction mean-square-error (MSE) as a function of the number of measurements for three different type of spectra. Above approximately 30 measurements, the quality of the reconstruction in terms of MSE is almost constant. Accordingly 36 structures are chosen.

Based on the design, the spectrometer is fabricated on a silicon-on-sapphire (SOS) wafer with a 500 nm Si layer. The 6×6 different patterns are defined using electron-beam lithography and then transferred to the silicon layer via reactive-ion etching (RIE) down to 150 nm. Each pattern consists of a periodic array of air holes (Supplementary Figure 2). Targeting at the spectral range from 550 nm to 750 nm, the hole diameters d range from 100 nm to 400 nm, the lattice constants P_1 and P_2 vary from 300 nm to 500 nm, and the angle θ between them varies from 30° to 90°. The chip is then flipped and placed on a CMOS sensor.

Supplementary Note 3: Characterization of the Angle Dependence of the Device

As shown in Supplementary Figure 3, the spectral response of our PC structures is angle dependent. To characterize the angle dependence of our PC spectrometer, the device was tested by measuring unknown spectra at different incident angles. Supplementary Figure 4 shows the measurement results for a signal generated by a combination of a lamp (ASBN-W) and a bandpass filter centered at 610 nm (Thorlabs, FB610). We use the same recovery algorithm as the one used for Figure 2 in the main text. For each angle, the spectral response of each PC structure is calibrated before measuring the unknown spectrum. Regardless of the incident angle, the spectrum can be obtained accurately, which indicates that the device sustains good performance for varying incident angles when calibration and signal measurement are performed at the same angle.

We also analyzed the cases when the unknown signal is measured at angles that are different from the calibration angle. Supplementary Figure 5 displays the test results for the measurements of two different spectra. For both cases, the incident beams are tilted by 1 to 4 degrees with

respect to the calibration angle. The device maintains good performance within a 1-degree shift. When the incident angle deviates more and more from the calibration angle, the recovered signals degrade while the main features persist.

Supplementary References

[1] M. Grant, S. Boyd, and Y. Ye, *Cvx: Matlab software for disciplined convex programming* (2008).

[2] M. C. Grant and S. P. Boyd, in *Recent advances in learning and control* (Springer, 2008), pp. 95–110.

[3] V. Liu and S. Fan, Computer Physics Communications **183**, 2233 (2012).

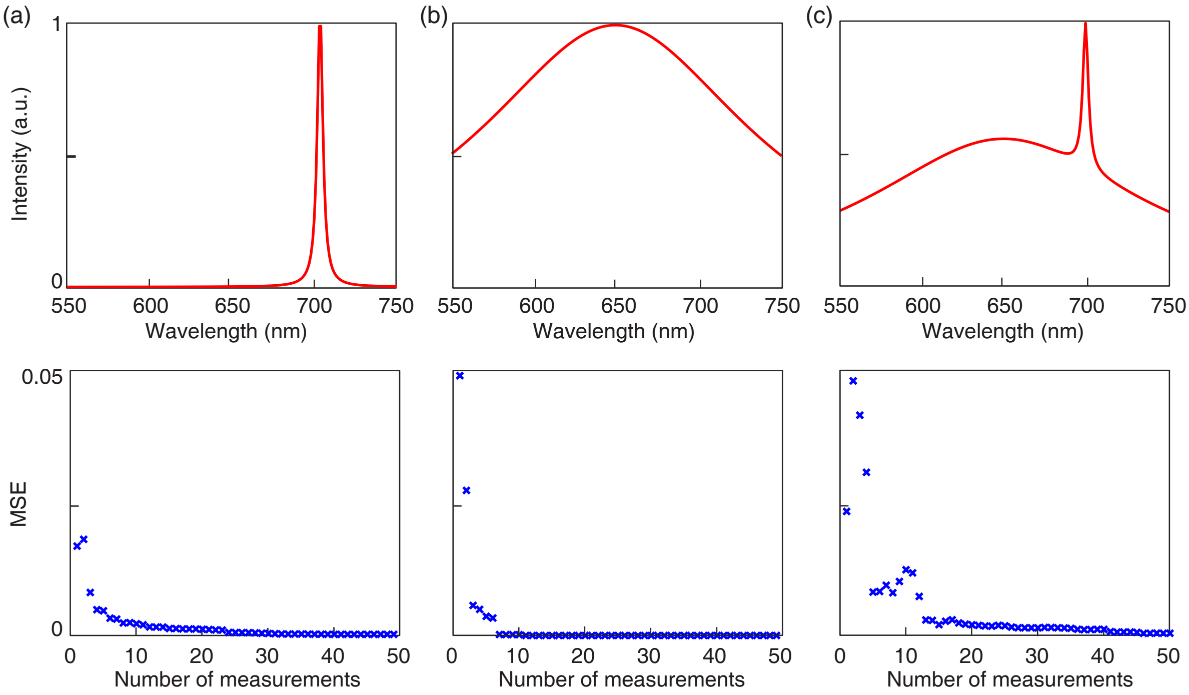

Supplementary Figure 1: **Reconstruction mean squared error (MSE) versus the number of measurements.** Top: three different types of spectra being tested: (a) a narrowband signal (b) a broadband signal, and (c) a broadband signal with a sharp peak, 201 non-zero unknowns. Bottom: reconstructed MSE versus the number of measurements for the signals shown in the top panel of (a)-(c), respectively.

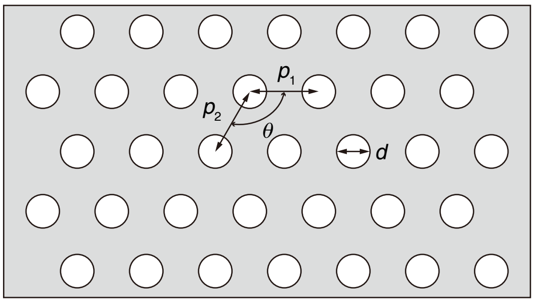

Supplementary Figure 2: **Top view of a representative PC structure.** Each of the 6×6 structure consists a periodic array of air holes, with varying hole size, lattice shape, and period to function as a unique spectral filter.

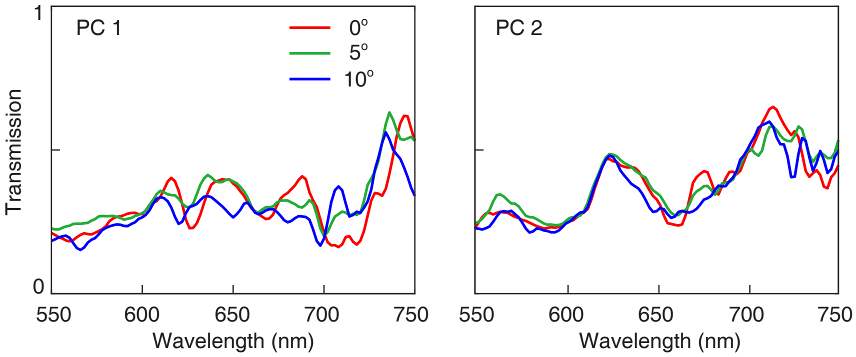

Supplementary Figure 3: **Angle dependence of PC structures.** Measured transmission spectrum $T(\lambda)$ for two particular PC structures when illuminated at different angles with unpolarized light.

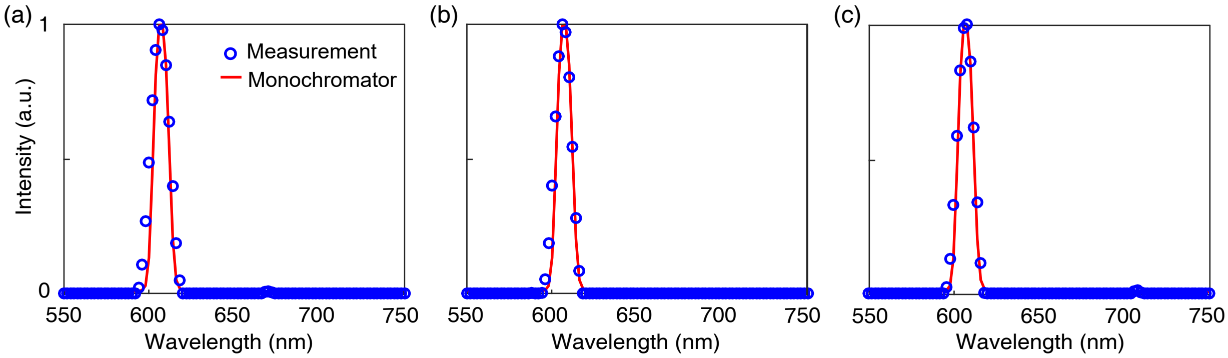

Supplementary Figure 4: **Angle dependency test of the PC spectrometer, when the calibration and signal measurements are performed at the same angle with unpolarized light.** (a)-(c) Incident angles are 5° apart. For all the three incident angles, the reconstructed spectra (blue circles) match well with the reference spectra (red solid line) obtained by a monochromator.

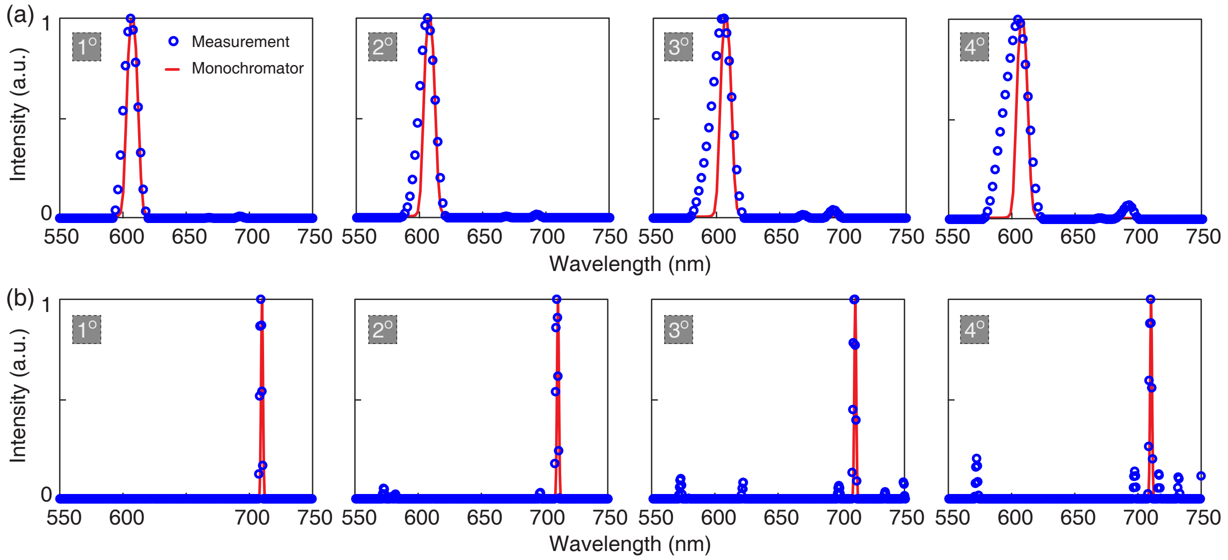

Supplementary Figure 5: **Angle dependency test of the PC spectrometer when the calibration and signal measurements are performed at different angles with unpolarized light.** The signal is incident at angles with 1°-4° shift (left to right) from the calibration angle. Measurements for (a) the for same incident spectrum as the one in Supplementary Figure 4. The measurements in (b) are for a narrowband spectrum 710 nm with a bandwidth of 1.4 nm.
